# The Preservative Sorbic Acid Targets Respiration, Explaining the Resistance of Fermentative Spoilage Yeast Species

**DOI:** 10.1128/mSphere.00273-20

**Published:** 2020-05-27

**Authors:** Malcolm Stratford, Cindy Vallières, Ivey A. Geoghegan, David B. Archer, Simon V. Avery

**Affiliations:** a School of Life Sciences, University of Nottingham, Nottingham, United Kingdom; University of Georgia

**Keywords:** weak acid preservatives, mitochondria, beverage spoilage, acetic acid, fungi, food preservation, food preservatives, food spoilage, respiration, yeasts

## Abstract

A small number (10 to 20) of yeast species cause major spoilage in foods. Spoilage yeasts of soft drinks are resistant to preservatives like sorbic acid, and they are highly fermentative, generating large amounts of carbon dioxide gas. Conversely, many yeast species derive energy from respiration only, and most of these are sorbic acid sensitive and so prevented from causing spoilage. This led us to hypothesize that sorbic acid may specifically inhibit respiration. Tests with respirofermentative yeasts showed that sorbic acid was more inhibitory to both Saccharomyces cerevisiae and Zygosaccharomyces bailii during respiration (of glycerol) than during fermentation (of glucose). The respiration-only species Rhodotorula glutinis was equally sensitive when growing on either carbon source, suggesting that ability to ferment glucose specifically enables sorbic acid-resistant growth. Sorbic acid inhibited the respiration process more strongly than fermentation. We present a data set supporting a correlation between the level of fermentation and sorbic acid resistance across 191 yeast species. Other weak acids, C_2_ to C_8_, inhibited respiration in accordance with their partition coefficients, suggesting that effects on mitochondrial respiration were related to membrane localization rather than cytosolic acidification. Supporting this, we present evidence that sorbic acid causes production of reactive oxygen species, the formation of petite (mitochondrion-defective) cells, and Fe-S cluster defects. This work rationalizes why yeasts that can grow in sorbic acid-preserved foods tend to be fermentative in nature. This may inform more-targeted approaches for tackling these spoilage organisms, particularly as the industry migrates to lower-sugar drinks, which could favor respiration over fermentation in many spoilage yeasts.

**IMPORTANCE** Spoilage by yeasts and molds is a major contributor to food and drink waste, which undermines food security. Weak acid preservatives like sorbic acid help to stop spoilage, but some yeasts, commonly associated with spoilage, are resistant to sorbic acid. Different yeasts generate energy for growth by the processes of respiration and/or fermentation. Here, we show that sorbic acid targets the process of respiration, so fermenting yeasts are more resistant. Fermentative yeasts are also those usually found in spoilage incidents. This insight helps to explain the spoilage of sorbic acid-preserved foods by yeasts and can inform new strategies for effective control. This is timely as the sugar content of products like soft drinks is being lowered, which may favor respiration over fermentation in key spoilage yeasts.

## Abstract (precis)

Spoilage by yeasts and molds is a major contributor to food and drink waste, which undermines food security. Weak acid preservatives like sorbic acid help to stop spoilage, but some yeasts, commonly associated with spoilage, are resistant to sorbic acid. Different yeasts generate energy for growth by the processes of respiration and/or fermentation. Here, we show that sorbic acid targets the process of respiration, so fermenting yeasts are more resistant. Fermentative yeasts are also those usually found in spoilage incidents. This insight helps to explain the spoilage of sorbic acid-preserved foods by yeasts and can inform new strategies for effective control. This is timely as the sugar content of products like soft drinks is being lowered, which may favor respiration over fermentation in key spoilage yeasts.

## INTRODUCTION

Foods and beverages may be spoiled by yeasts and molds despite the use of preservatives such as sorbic acid. Sorbic acid and acetic acid are both weak acids that inhibit many yeasts effectively, but weak-acid-resistant species can still cause contamination. Spoilage yeasts are a very small proportion of the overall numbers of known species. The number of recognized yeast species is in excess of 1,500 (1), while those yeasts causing the majority of spoilage cases are limited to around 12 species (2). Similarly, only a small proportion of known mold species cause spoilage (3). Food lost to spoilage is a major food security concern (4).

Complaints by consumers can be due to visible contamination, off-flavors, or products being “blown,” or exploding in sealed packaging. This is caused by spoilage yeasts generating high levels of carbon dioxide through fermentation and can result in physical injury (5). Yeast species found in factories producing foods or drinks have been characterized as belonging to Group 1, 2, or 3, according to incidence and spoilage risk (6). Zygosaccharomyces bailii is among a small number of spoilage yeasts categorized in Group 1. The Group 1 yeasts tend to be highly fermentative and show marked resistance to preservatives and osmotic stress. It is probable that most yeast species that have been recorded as fermentative (1) will also use respiration in different circumstances. In Saccharomyces cerevisiae, high levels of glucose suppress respiration and the glucose is fermented (7). Respiration arises at lower glucose levels (≤0.5% to 1%, wt/vol). Furthermore, many yeast species are recorded as being nonfermentative, generating energy from carbon sources using respiration only. Approximately two-thirds of all yeast species grow by respiration only (8). The Group 3 yeasts are very largely respiratory, and these species are sensitive to sorbic acid (6).

Many preservatives are weak acids, including propionic acid, sulfite (SO_2_), benzoic acid, and sorbic acid. It has previously been indicated that a major effect of these preservatives is acidification of the yeast cytoplasm (9–12). The weak acids enter cells rapidly by diffusion and can flow freely in and out (11, 13). As weak acids arrive in the yeast cytoplasm at neutral pH, they spontaneously dissociate to the anion, e.g., acetate, or bisulfite ion, and release H^+^. Large concentrations of weak acids can generate high levels of H^+^ and thereby lower the cytoplasmic pH, affecting protein structure and function (14). However, different weak acids inhibit yeasts at different levels; acetic acid requires ∼120 mM to inhibit S. cerevisiae, while sorbic acid inhibits at ∼3 mM. At these concentrations, acetic acid markedly lowers the internal pH (pH_i_) but sorbic acid causes only a very minor lowering of pH_i_ (11, 15). Therefore, the primary inhibition mechanism for sorbic acid and other similar weak acids (hexanoic acid, heptanoic acid, octanoic acid) has yet to be demonstrated. Partial inhibition of glycolysis rather than via pH_i_ has been suggested (15). Moreover, oil/water partition coefficients indicate that acetic acid should largely remain soluble in water whereas sorbic acid, which is more lipophilic, should largely occupy membranes. Certain membrane proteins have been shown to be specifically inhibited by sorbic acid (16, 17).

In the present study, based on a potential membrane action of sorbic acid and the observation that most sorbic-resistant spoilage yeasts grow by fermentation, we hypothesized, and then confirmed, that sorbic acid preferentially inhibits respiration over fermentation. A mechanistic explanation was then sought at the level of inhibition of mitochondrial function by sorbic acid. This insight should open a new avenue for understanding modes of weak-acid action and provide a new rationale for food preservation.

(This article was submitted to an online preprint archive [18].)

## RESULTS

### Sorbic acid sensitivity of respiratory growth in spoilage yeasts.

**FIG 1:**
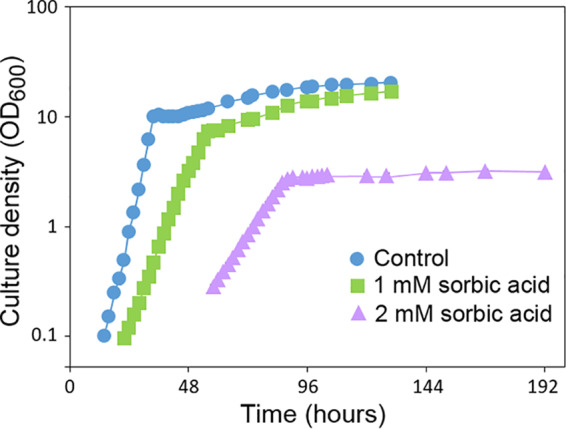
Effect of sorbic acid on growth of S. cerevisiae with glucose. S. cerevisiae Δ*pad1* was cultured with shaking at 120 rpm and 24°C in flasks containing YEPD, pH 4.0, supplemented with the indicated concentrations of sorbic acid. Error bars (SD, *n* = 3) were smaller than the dimensions of the symbols.

We examined the effect of sorbic acid on growth of the model and food spoilage yeast S. cerevisiae. As S. cerevisiae can decarboxylate sorbic acid, which requires the *PAD1* gene (19), an S. cerevisiae Δ*pad1* deletant (MIC 3 mM sorbic acid, pH 4.0) was used for these growth experiments in order to prevent degradation of the weak acid during the experiment (such degradation enables later outgrowth, complicating discrimination of the normal growth phases). S. cerevisiae Δ*pad1* grew exponentially (∼2-h doubling time) in yeast extract-peptone-dextrose (YEPD; pH 4.0), up to an optical density at 600 nm (OD_600_) of ∼10 (Fig. 1), and thereafter more slowly, consistent with respiration of ethanol (7). Addition of 1 mM sorbic acid reduced the growth rate, to give a doubling time of ∼4.5 h while 2 mM sorbic acid increased the doubling time to approximately 8.5 h (Fig. 1). The growth yield at the end of the exponential growth phase was also decreased by the addition of sorbic acid, with an OD_600_ of ∼7.5 achieved in 1 mM sorbic acid and an OD_600_ of ∼3.0 in 2 mM sorbic acid. At 2 mM sorbic acid, the subsequent, slow respiratory phase of growth appeared to be inhibited.

**FIG 2:**
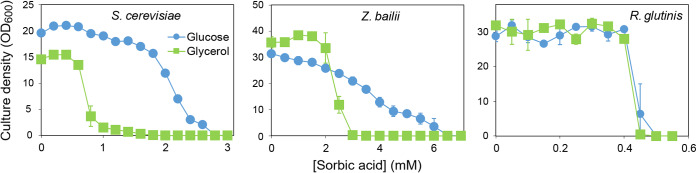
Growth inhibition by sorbic acid in yeasts cultured with glucose or glycerol. S. cerevisiae, Z. bailii, or *R. glutinis* was cultured in either 3% (wt/vol) glucose or 3% glycerol, in YEP (pH 4.0) supplemented with the indicated concentrations of sorbic acid. OD_600_ in flasks was determined after shaking at 120 rpm, 24°C, for 14 days. Points are means from three replicate determinations ± SD.

Since the respiratory phase of S. cerevisiae growth appeared to be stopped at 2 mM sorbic acid (Fig. 1), it was hypothesized that sorbic acid may selectively inhibit respiratory growth, so this was compared specifically by cultivation in YEP pH 4.0 supplemented with 30 g/liter glycerol (no glucose). With 30 g/liter glucose, growth was particularly inhibited at sorbic acid levels in excess of 2 mM sorbic acid, dropping to zero growth at the MIC of 3 mM sorbic acid (Fig. 2). In 30 g/liter glycerol, full growth was limited to sorbic acid concentrations of ≤0.7 mM and the MIC was 1.8 mM sorbic acid. This indicated that respiratory growth of S. cerevisiae is hypersensitive to sorbic acid. A similar test was carried out with 30 g/liter ethanol, an alternative respiratory substrate for S. cerevisiae, and again growth was inhibited at ∼1.5 mM sorbic acid (data not shown).

Respiratory growth was also tested with the spoilage yeast Z. bailii in 30 g/liter glycerol and compared to growth in 30 g/liter glucose (Fig. 2) (previously, Z. bailii has been reported to grow in glycerol, ethanol, or acetic acid [20]). Z. bailii grew well in both 30 g/liter glucose and 30 g/liter glycerol. However, the sorbic acid MIC in glycerol was 3.1 mM whereas in glucose the MIC was 6.6 mM sorbic acid. Therefore, both Z. bailii and S. cerevisiae were inhibited at lower levels of sorbic acid when growing by respiration.

A similar experiment was carried out with Rhodotorula glutinis, which has been reported as fermentation deficient in glucose and other sugars (1, 8). This red yeast grew similarly well in either 30 g/liter glucose or 30 g/liter glycerol in the shaking flasks (Fig. 2). However, *R. glutinis* was hypersensitive to sorbic acid, and the MIC was almost identical at ∼0.5 mM acid whether in 30 g/liter glucose or 30 g/liter glycerol. As sorbic acid resistance in glucose (seen with S. cerevisiae or Z. bailii) was absent in this respiration-only yeast, the results further corroborate that sorbic acid selectively inhibits respiratory growth.

### Relationship between sorbic acid sensitivity and respiratory or fermentative growth of diverse yeast species.

Above, S. cerevisiae, Z. bailii, and *R. glutinis* had all been grown in 30 g/liter glucose or 30 g/liter glycerol during treatment with sorbic acid. To ascertain whether the key observations with these species extended to other yeasts, a further 14 species were tested in a similar way, as representatives of Davenport Groups 1, 2, and 3 (Table 1). On glucose, as expected, Group 1 yeasts were highly resistant to sorbic acid, Group 2 were moderately resistant, and Group 3 were sensitive to sorbic acid (Table 1). All four species in Group 3 exhibited similar sensitivity to sorbic acid whether in 30 g/liter glucose or 30 g/liter glycerol. As indicated above for *R. glutinis*, these species were all fermentation defective in glucose (see Table S3 in the supplemental material) so can be assumed to be respiring in both glucose and glycerol. Within Group 2, three of the test species show moderate fermentation (Wickerhamomyces anomalus, Candida pseudointermedia, Candida parapsilosis) while two have high fermentation (Saccharomyces cerevisiae and Torulaspora delbrueckii) (Table S3). The MICs of sorbic acid for these yeasts in the respiratory substrate glycerol were ∼60% to 90% of the MICs observed in glucose (Table 1). This indicated that fermentative metabolism by the Group 2 yeasts was associated with a moderate elevation of sorbic acid resistance. Sorbic acid resistance of the high-fermentation Group 1 yeasts tended to be the most strongly affected by carbon source. The sorbic acid MIC for these species when growing by respiration was between 40% and 65% of their MICs when growing by fermentation.

**TABLE 1:** Respiratory growth sensitizes spoilage species to sorbic acid

Group^*a*^	Strain	Yeast species	Sorbic acid MIC (mM)		Glycerol/glucose MIC ratio (%)
			Glucose	Glycerol	
3	628	*Cryptococcus magnus*	0.37^*b*^	0.36	97
3	95	Rhodotorula mucilaginosa	0.425	0.4	94
3	92	*Rhodotorula glutinis*	0.48	0.46	96
3	546	*Cryptococcus laurentii*	0.81	0.76	94
2	NCYC 3371	*Wickerhamomyces anomalus*	1.35	1.25	93
2	519	Candida pseudointermedia	1.45	1.15	86
2	69	Candida parapsilosis	2.5	1.85	74
2	529	Torulaspora delbrueckii	3	1.75	58
2	BY4741	Saccharomyces cerevisiae	3	1.8	60
2	BY4741 Δ*pad1*	Saccharomyces cerevisiae	3	1.8	60
2	BY4741 petite	Saccharomyces cerevisiae	3	0	
1	NCYC 3297	*Candida pseudolambica*	3.8	2.5	65
1	55	Kazachstania exigua	4	1.6	40
1	522	*Pichia kudriavzevii*	4.2	2.3	55
1	NCYC 1555	Zygosaccharomyces *bisporus*	4.7	2.4	51
1	NCYC 1766	Zygosaccharomyces bailii	6.6	3.1	47
1	NCYC 2789	Zygosaccharomyces *lentus*	6.2	3.2	52

a Davenport grouping according to spoilage incidence (6).

b Sorbic acid MIC, mean from triplicate determinations after 14 days of shaking at 120 rpm, 24°C, in flasks containing YEP, pH 4.0, supplemented with sorbic acid and 30 g/liter of either glucose or glycerol.

**FIG 3:**
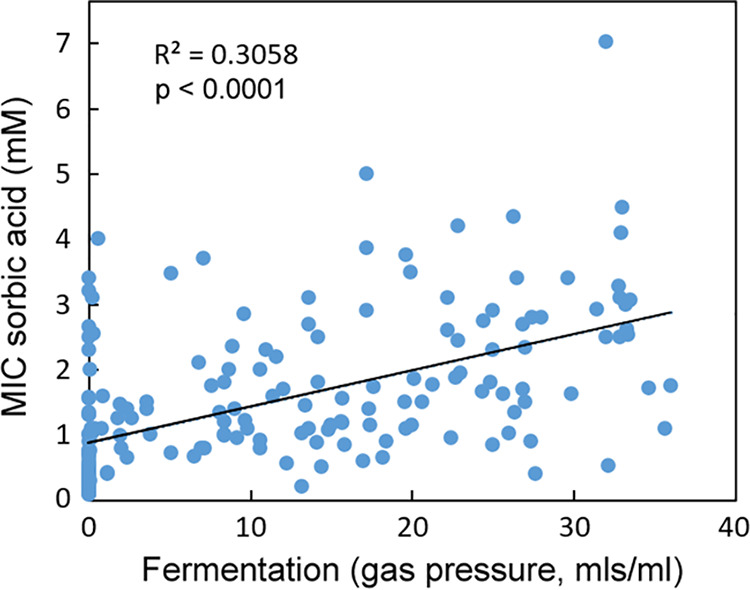
Comparison of the levels of fermentation and sorbic acid resistance exhibited by 191 yeast species (encompassing 687 yeast strains). Organisms are listed in Table S2. Sorbic acid resistance (MIC) was determined in YEPD (pH 4.0) after 14 days at 25°C, and the fermentation was tested after 28 days in static bottles in YEP with 180 g/liter (1 M) glucose. Fifty-three species showed zero fermentation. The slope was fitted by linear regression. *R*^2^ and *P* values (shown on the figure) were determined by Pearson correlation.

In glucose, there was overlap in the fermentation rates of the Group 1 and 2 yeasts (Table S3) but not in their MICs (Table 1). Therefore, a high level of fermentation alone did not appear to be sufficient to explain the highest levels of sorbic acid resistance. To test more rigorously any relationship between fermentation activity and sorbic acid resistance, we tested 687 yeast strains, representing 191 yeast species (Table S2), for sorbic acid MICs and fermentation level in 180 g/liter glucose (this higher glucose level accentuates differences in fermentation capacity [Table S3]). The data for different strains of each yeast species were averaged before plotting. There was a weak but significant positive correlation between fermentation and sorbic acid resistance (correlation, *R*^2^ = 0.3058; *P* < 0.0001) (Fig. 3). The correlation was a little weaker but remained significant when nonfermentative yeasts were excluded from the analysis (correlation, *R*^2^ = 0.2038; *P* < 0.0001). The bulk of the 53 species that showed no fermentation were found to be sensitive to sorbic acid, with just a few showing moderate resistance (e.g., Yarrowia lipolytica). The species with the greatest resistance tended to be those with the highest fermentation.

### Selective inhibition of respiratory activity by sorbic acid in yeasts.

**FIG 4:**
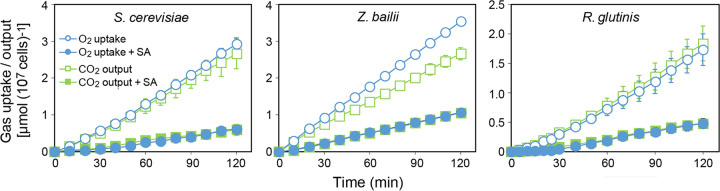
Inhibition of respiration by sorbic acid. Yeasts growing with shaking at 24°C in YEP (pH 4.0) containing 3% (wt/vol) glycerol were monitored for O_2_ uptake and CO_2_ output by Warburg manometry. Where indicated (closed symbols), sorbic acid (SA) was included at 1.8 mM for S. cerevisiae, 3.5 mM for Z. bailii, or 0.46 mM for *R. glutinis*. Points are means from three replicate determinations ± SD.

As relative dependence on respiration for growth should increase with decreasing fermentation, and respiration-only species were the most sorbic acid sensitive, we reasoned that respiratory metabolism could be a target of sorbic acid. About two-thirds of yeast species are recorded as nonfermentative (8) and so considered respiration-only species. We tested whether respiration is inhibited by sorbic acid using S. cerevisiae growing in 30 g/liter glycerol, using Warburg manometry. The data substantiated that the yeast was respiratory under these conditions, absorbing oxygen and producing equivalent carbon dioxide (Fig. 4). Inclusion of sorbic acid at the MIC level (1.8 mM) strongly inhibited respiration. After 120 min, oxygen removal and carbon dioxide production in the presence of sorbic acid were ∼20% of the control level. In the case of the major spoilage yeast Z. bailii, respiratory growth in 30 g/liter glycerol was associated with slightly higher oxygen absorption than carbon dioxide production, but these parameters were reduced to a similar level at 3.5 mM sorbic acid (approximating the relevant MIC for this organism). The experiment was repeated with the respiration-only yeast *Rhodotorula glutinis* in glycerol (Fig. 4). Sorbic acid at the relevant MIC (0.46 mM) inhibited the respiration of *R. glutinis*, to a similar extent as observed in S. cerevisiae and Z. bailii.

**FIG 5:**
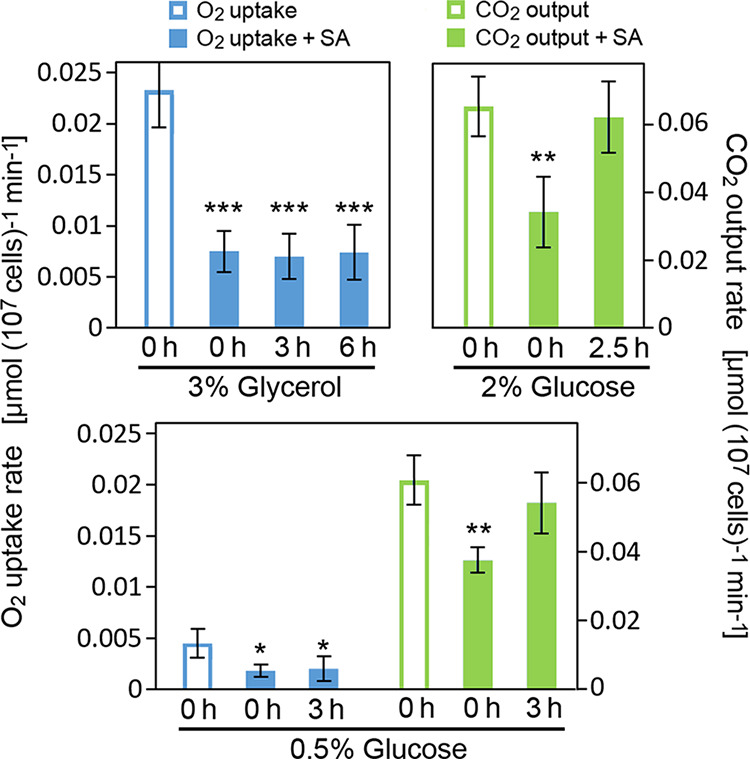
Comparative inhibition of respiration and fermentation by sorbic acid. S. cerevisiae growing with shaking at 24°C in YEP (pH 4.0) containing either glucose or glycerol at the indicated concentrations (wt/vol) was either treated or not with 1.6 mM sorbic acid (SA) and monitored for O_2_ uptake and CO_2_ output. Measurements were made for up to 40 min either immediately following sorbic acid treatment (0 h) or after the later exposure times indicated. Mean data are shown from four replicate determinations ± SD. *, *P* < 0.05; **, *P* < 0.01; ***, *P* < 0.001, according to Student’s *t* test, two-tailed.

The relative sorbic acid sensitivities of respiration and fermentation were compared in S. cerevisiae. During culture in glycerol and exposure to sorbic acid (1.6 mM), respiration was inhibited by ∼68% and did not recover over longer treatment times up to 6 h (Fig. 5). The fermentation rate during culture with 2% glucose was less strongly inhibited, by ∼48%, immediately following sorbic acid addition. Furthermore, fermentation quickly recovered to pretreatment levels after 2.5-h exposure to sorbic acid. This comparison was further tested at 0.5% glucose, a concentration that allows both fermentation and respiration in S. cerevisiae BY4741. Again, sorbic acid inhibited respiration by ∼60%, and this did not recover after 3 h, whereas there was less (∼38%) inhibition of fermentation, which largely recovered after 3 h (Fig. 5). The results indicate that respiration is more sensitive than fermentation to inhibition by sorbic acid.

### Selective inhibition of respiratory growth by different weak acids is correlated with chain length and membrane solubility.

**FIG 6:**
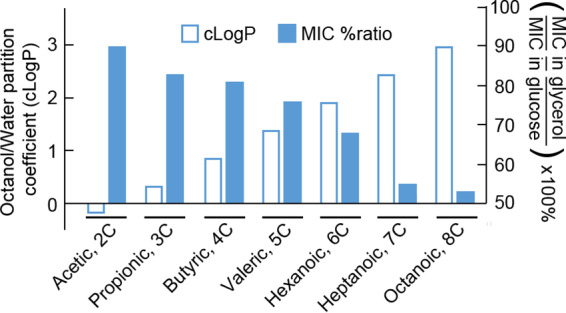
Membrane-partitioning tendency of acids correlates with relative toxicities to respiring S. cerevisiae. Determined after 14 days of shaking at 120 rpm, 24°C, in flasks containing YEP, pH 4.0, supplemented with weak acid and 30 g/liter of either glucose or glycerol. Underlying values are given in Table S4.

We also examined acetic acid, as a weak acid that is more hydrophilic (less membrane soluble) than sorbic acid. Much higher concentrations of acetic acid were required to inhibit yeast growth than sorbic acid. Moreover, unlike with sorbic acid, inhibition of S. cerevisiae by acetic acid was similar in 30 g/liter glycerol or 30 g/liter glucose, with MICs close to 140 mM (Fig. S1). This outcome was reflected also with Z. bailii, which was highly resistant to acetic acid in glycerol and in glucose, where the MIC levels were similar at ∼450 mM acetic acid (Fig. S1). To explore further potential relationships between weak-acid sensitivity of respiratory growth and weak-acid hydrophobicity, a wider range of weak acids with two- to eight-carbon lengths was tested in S. cerevisiae. MICs were determined for each weak acid during growth in both glycerol and glucose; the ratio between these MIC values provided an indication of the relative sensitivity of respiratory growth to each weak acid. In both media, the MICs declined markedly with increasing carbon chain length of the weak acids (Table S4). However, the relative decline in MIC differed in glycerol versus glucose. As a result, there was a tight inverse relationship between carbon chain length and relative inhibition of respiratory versus fermentative growth by the weak acids (Fig. 6). The longer-chain acids have a relatively high octanol/water partition coefficient (cLogP), predictive of greater membrane localization. Acetic acid is relatively hydrophilic and gave similar MICs during respiration (in glycerol) and fermentation (glucose), whereas the MICs of C_6_ (hexanoic) and C_8_ (octanoic) acids in glycerol were only 68% and 53% of those in glucose, respectively.

10.1128/mSphere.00273-20.1 FIG S1 Growth on glucose or glycerol in the presence of acetic acid. S. cerevisiae (top panel) or Z. bailii (bottom panel) was cultured in either 30 g/liter glucose (open squares) or 30 g/liter glycerol (closed squares), in YEP (pH 4.0) supplemented with the indicated concentrations of acetic acid. OD_600_ in flasks was determined after shaking at 120 rpm, 24°C, for 14 days. Points are means from three replicate determinations ± SD. Download FIG S1, TIF file, 0.2 MB. Copyright © 2020 Stratford et al. 2020 Stratford et al. This content is distributed under the terms of the Creative Commons Attribution 4.0 International license.

### Mechanisms underlying sorbic acid sensitivity of yeast respiration.

**FIG 7:**
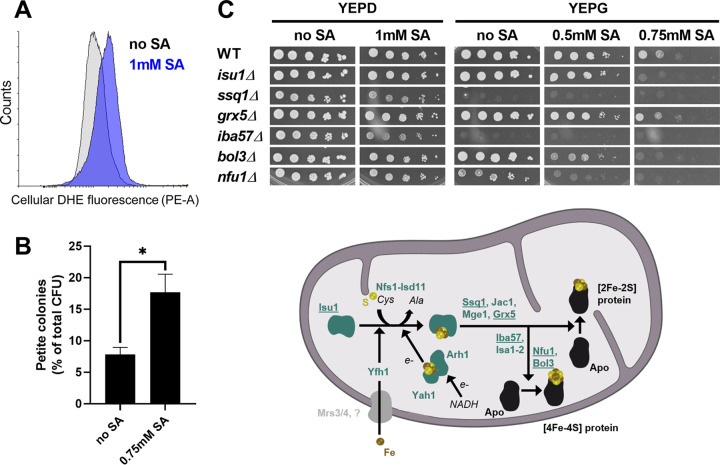
ROS production and damage to mitochondrial function with sorbic acid during respiration. (A) ROS production upon sorbic acid treatment was measured using the fluorescent probe DHE after 4 h of incubation of S. cerevisiae with sorbic acid. (B) S. cerevisiae colonies cultivated for at least 7 days with or without 0.75 mM sorbic acid (i.e., subinhibitory concentration) were replica plated onto YEP-glucose (YEPD) and YEP-glycerol (YEPG) to assess petite-cell formation (petite cells do not grow on YEPG). Mean data are shown from triplicate independent growth experiments ± SEM. *, *P* < 0.05 according to Student’s *t* test, two-tailed. (C) Upper panel: serial dilutions of S. cerevisiae BY4743 and the indicated isogenic deletion strains were spotted onto agar plates containing a fermentable (YEPD) or respiratory (YEPG) carbon source with or without sorbic acid and incubated at 30°C for 2 or 4 days, respectively. Lower panel: simplified scheme showing proteins involved in the biogenesis of FeS clusters and their transfer to mitochondrial apoproteins. Underlined proteins correspond to deletants examined.

The above data collectively support the hypothesis that longer-chain-length weak acids, like sorbic acid, selectively target respiration by yeasts and that this effect is correlated with membrane solubility. Membrane perturbation and effects on the respiratory chain are commonly associated with production of reactive oxygen species (ROS) (21, 22). In the present study, analysis using the ROS probe dihydroethidium (DHE) indicated that sorbic acid, at subinhibitory concentrations, promotes ROS production in S. cerevisiae (Fig. 7A). One consequence of mitochondrial ROS production in organisms like S. cerevisiae is the formation of petite (mitochondrion-defective) cells, arising from mitochondrial DNA damage; mitochondrial DNA encodes mainly proteins that are part of the respiratory chain (23). We tested whether petite-cell formation could be a contributor to the apparent selective targeting of respiration during sorbic acid treatment. We grew S. cerevisiae on YEPD agar supplemented or not with sorbic acid and then assayed for respiratory competency by replica plating colonies to YEP-glycerol (YEPG) agar. There was an ∼2.3-fold increase in petite-cell frequency in the presence of 0.75 mM sorbic acid compared to control (Fig. 7B). ROS are also known to impair iron-sulfur cluster (ISC) biogenesis, which takes place in the mitochondria. We tested whether mutants of the ISC biogenesis pathway were hypersensitive to sorbic acid treatment. On glycerol, the *bol3Δ* and *nfu1Δ* mutants and to some extent the *isu1Δ* mutant were more sensitive to the weak acid than the wild-type strain, suggesting an effect of sorbic acid on iron-sulfur (FeS) protein formation (Fig. 7C). Isu1 is a scaffold protein involved in the first step of [2Fe-2S] cluster assembly while Nfu1, assisted by Bol3, facilitates the transfer of [4Fe-4S] clusters from the assembly complex to client proteins while protecting the cofactor from oxidative damage (24) (Fig. 7C). Succinate dehydrogenase, essential for respiration, is one of these client proteins. The collective data suggest that sorbic acid generates ROS in mitochondria, which could lead to depletion of functional respiratory complexes through pathways including petite-cell formation and FeS cluster targeting.

## DISCUSSION

This study shows that respiration by yeasts is more sensitive than fermentation to inhibition by the major food preservative sorbic acid. Furthermore, fermentative metabolism shows greater recovery than respiratory metabolism over time after sorbic acid shock. This is an important result for industry and helps explain why the highly fermentative species such as Z. bailii can cause such catastrophic spoilage incidents. Indeed, we show that historical categorization of yeast species according to their propensity to cause food or beverage spoilage (6) maps closely to their capacities to ferment or respire, with the yeasts least associated with spoilage (Group 3) being fermentation defective and most reliant on respiration.

Respiration in glycerol was shown to be inhibited by sorbic acid in S. cerevisiae, and 14 other yeast species showed sorbic acid-sensitive respiratory growth. In addition, a screen of 191 yeast species established a correlation between fermentative activity and sorbic acid resistance. Previous tests carried out on a mold, Aspergillus niger, had shown that sorbic acid inhibited respiration and germination in asexual spores (25). However, fermentation is low or absent in such filamentous fungi, precluding the type of comparisons established here with yeasts. Respiratory growth was inhibited by sorbic acid at 3.1 mM for Z. bailii (Group 1), 1.8 mM for S. cerevisiae (Group 2), and 0.46 mM for *R. glutinis* (Group 3), but these relative differences were accentuated in glucose, where growth can occur by fermentation in Z. bailii and S. cerevisiae.

Other weak acids also inhibited respiratory growth in S. cerevisiae (see Table S4 in the supplemental material), and the toxic effects of the different weak acids were much greater with the longer-chain weak acids. This strongly suggests that the relative toxic effect is related to lipophilicity and the weak acid being absorbed into lipid membranes. A weak acid such as decanoic acid (10 carbons) is absorbed so profusely into the membranes that the membranes burst, causing rapid cell death (26). Previous studies have indicated that yeast respiration can be inhibited by certain weak acids (27–29). However, such earlier work typically has not distinguished whether these are selective (causative) effects, targeting respiration, as opposed to broader inhibition of cell activities by sorbic acid, among which decreased respiration would be just one of several effects. The ability to dissect respiratory from fermentative growth in yeasts provided a valuable tool to resolve these possibilities in this study.

How does sorbic acid target respiration? To help address this question, we initially had attempted a screen of the yeast homozygous deletant collection (30, 31) to find gene functions required for respiratory sensitivity to sorbic acid. Although a number of deletants on glycerol did exhibit higher sorbic acid resistance than the wild type, including an *atg9* deletant defective in autophagy, the identified functions did not suggest an obvious pathway required for sorbic acid sensitivity (Table S5). Instead, we considered the correlation between the respiratory inhibition and membrane solubilities of the different weak acids (above) and the ROS production that is commonly associated with mitochondrial-membrane perturbation (21, 22). Our results showed that sorbic acid can promote ROS production. Furthermore, key effects of ROS on cellular function (32–34) could be seen in sorbic acid-treated cells, i.e., formation of mitochondrion-defective petite cells and indications of FeS cluster pathway defects. The mitochondrial DNA damage that typically produces petite cells is normally irreversible. Therefore, the increased incidence of petite cells could help to explain the nonrecovery of respiratory activity that we observed following sorbic acid treatment, whereas fermentation (which can continue in petite cells) did recover. It was also notable that specific FeS pathway functions which conferred sorbic acid resistance are involved in shielding FeS delivery from ROS, to client proteins like succinate dehydrogenase, which is essential for respiration. We propose a model in which ROS generated by membrane-localized sorbic acid causes depletion of mitochondrial respiratory function, through pathways including petite-cell formation and FeS cluster targeting.

It has been argued that population heterogeneity (preservative heteroresistance) among individual cells or spores within spoilage-yeast or -mold populations has major implications for food spoilage (35–38). Accordingly, it may take only a few, preservative-hyperresistant cells to initiate spoilage. It was previously reported that Z. bailii causes spoilage at a level of contamination of one cell/bottle (39). Although heterogeneity was not a focus of this study, our finding that mitochondrial function is a key determinant of sorbic acid sensitivity is notable in this context. That is because mitochondrial activity and cellular redox status are quite heterogeneous within cell populations (40, 41). In the present study, petite-cell formation with sorbic acid was not uniform across the yeast cell populations. Therefore, it is possible that mitochondrial heterogeneity could be one factor determining the preservative heteroresistance of spoilage yeasts, the mechanistic bases for which have yet to be resolved.

Overall, this paper shows that respiration is selectively inhibited by sorbic acid, seemingly in a wide range of yeast species. We propose that ROS generated by membrane-localized weak acid could explain this respiratory targeting. These findings are especially timely as the beverages industry seeks to reduce the sugar content of its products, in response to soft-drink sugar taxes introduced by a number of governments. Because of catabolite repression, as glucose is decreased, respiratory activity of spoilage yeasts increases (assuming there is available oxygen) (7). Our results suggest that an increased reliance of spoilage yeasts on respiration for growth in lower-sugar beverages is likely to strengthen the preservative efficacy of weak acids. In contrast, there is evidence that sugar-substituted foods can be more prone to spoilage by molds (42), organisms that are more reliant on respiration regardless of glucose concentration. It seems that the profile of organisms associated with spoilage incidents is likely to change as the glucose content of soft drinks is lowered.

## MATERIALS AND METHODS

### Yeast species and strains.

The principal yeast species and strains used in this study are listed in Table S1 in the supplemental material, which records both previous (8) and updated (1) strain numbers and species names, and the sources of the yeasts. In addition, 687 yeast strains spanning 191 species were tested for the relationship between fermentation and sorbic acid resistance, and these are listed in Table S2. The identities of all the strains were determined by sequencing the D1/D2 region of the 26S ribosomal DNA (rDNA) (43). Experiments with S. cerevisiae were with strain BY4741 unless specified otherwise. Yeasts were stored in glycerol on ceramic beads at −80°C (Microbank) and maintained in the short term on malt extract agar (MEA; Oxoid) slopes at 4°C.

10.1128/mSphere.00273-20.2 TABLE S1 Principal yeast species used in this research. Original and current species names are provided (1) together with the strain sources. Download Table S1, DOC file, 0.06 MB. Copyright © 2020 Stratford et al. 2020 Stratford et al. This content is distributed under the terms of the Creative Commons Attribution 4.0 International license.

10.1128/mSphere.00273-20.3 TABLE S2 Full list of yeast species, encompassing 687 strains, which were tested for fermentation and sorbic acid MIC (results summarized in Fig. 3). Download Table S2, DOC file, 0.5 MB. Copyright © 2020 Stratford et al. 2020 Stratford et al. This content is distributed under the terms of the Creative Commons Attribution 4.0 International license.

10.1128/mSphere.00273-20.4 TABLE S3 Fermentation by spoilage and nonspoilage yeast species. Download Table S3, DOC file, 0.06 MB. Copyright © 2020 Stratford et al. 2020 Stratford et al. This content is distributed under the terms of the Creative Commons Attribution 4.0 International license.

10.1128/mSphere.00273-20.5 TABLE S4 Resistance of S. cerevisiae to weak acids with different carbon chain lengths. Download Table S4, DOC file, 0.05 MB. Copyright © 2020 Stratford et al. 2020 Stratford et al. This content is distributed under the terms of the Creative Commons Attribution 4.0 International license.

10.1128/mSphere.00273-20.6 TABLE S5 Gene functions contributing to sorbic acid sensitivity of respiratory growth on glycerol. Download Table S5, DOC file, 0.07 MB. Copyright © 2020 Stratford et al. 2020 Stratford et al. This content is distributed under the terms of the Creative Commons Attribution 4.0 International license.

### Growth conditions.

The growth medium for routine culturing was YEPD, containing 20 g/liter glucose, 20 g/liter bacteriological peptone (Oxoid), and 10 g/liter yeast extract (Oxoid). The medium was adjusted to pH 4.0 with 5 M HCl prior to sterilization by autoclaving. Where specified, the glucose concentration was amended to 5, 10, 30, or 180 g/liter. Other experiments required yeast extract and peptone (YEP), with 30 g/liter glycerol adjusted to pH 4.0. Some batches of peptone or yeast extract contained low levels of glucose, so these were avoided when zero or low levels of known amounts of glucose were required.

Starter cultures were grown in 10 ml YEPD (pH 4.0) in 28 ml McCartney bottles, inoculated with yeast from MEA slopes. Bottles were incubated statically for 48 h at 24°C. These starter cultures were used to inoculate experimental cultures, which comprised either 5 ml or 10 ml YEPD, pH 4.0, in 28-ml McCartney bottles, or 40 ml YEPD, pH 4.0, in 100-ml conical flasks which were shaken at 120 rpm, 24°C.

Sorbic acid was dissolved in methanol to make stock solutions of 100, 200, or 400 mM. Aliquots from the stock solution appropriate for the desired final sorbic acid concentration were transferred to the medium before the pH 4.0 adjustment. At pH 4.0, ∼85% of added sorbic acid exists as free acid and ∼15% as anion. Acetic acid and propionic acids (liquids) were added directly to media before adjusting the pH to 4.0. Butyric acid, valeric acid, hexanoic acid, heptanoic acid, and octanoic acid (liquids) were diluted in methanol. The final methanol levels in media did not exceed 1% (vol/vol) (20% to 30% methanol is required to exert toxicity in yeast).

### Measurement of gas pressure from fermentation.

Fermentation by yeasts uses sugars, commonly glucose, to generate ATP accompanied by metabolism of sugar to ethanol and CO_2_ (1, 8). The level of fermentation was estimated by measuring the gas pressure generated by the yeast. Yeasts were inoculated into 10 ml YEPD (pH 4.0), in sealed triplicate 28-ml McCartney bottles, and incubated at 24°C for up to 28 days. Glucose was routinely used at 20 g/liter, but 5, 10, and 30 g/liter were also tested. Pressure in the sealed bottles was tested at 28 days with a gas syringe (average gas volumes were between 0 and 30 ml, when adjusted to atmospheric pressure). A parallel fermentation was carried out using 180 g/liter glucose (1 M), in 5 ml YEPD (pH 4.0) in the sealed bottles. Gas volumes up to 200 ml were obtained in the 28-ml McCartney bottles (approximately 10 atmospheres). Gas pressure was calculated as the volume (milliliters) of gas generated per 1 ml of medium.

### Warburg manometry.

A Warburg manometer was used to measure the absorption of O_2_ and the efflux of CO_2_ from yeast, by determining gas pressures over a period of 70 min. The shaking flasks contained 3 ml of medium and either 0.4 ml of water or 20% KOH held at 24°C. For measuring respiration, glucose-free YEP (pH 4.0) medium was supplemented with glycerol at 30 g/liter and yeasts were precultivated in this medium for 12 to 14 h at 24°C and then transferred to fresh medium at 10^7^ cells/ml prior to measurements in the manometer. Respiration was confirmed as the sole metabolic route where absorption of oxygen and efflux of carbon dioxide were almost identical. Fermentation (carbon dioxide efflux but no absorption of oxygen) was tested in medium supplemented with glucose (20 g/liter) rather than glycerol, after precultivation in this medium for 12 to 14 h at 24°C before transfer to fresh medium at 10^7^ cells/ml. A lower glucose concentration (5 g/liter) was used to assess both respiration and fermentation.

### ROS accumulation.

Detection of cellular reactive oxygen species (ROS) was with the fluorescent probe dihydroethidium (DHE) (44). Samples of yeast culture (S. cerevisiae BY4743) grown in YEPD, (pH 4.0) with or without sorbic acid for 4 h were centrifuged, washed, and then incubated in 100 μl phosphate-buffered saline (PBS) with 5 μM DHE for 30 min at 30°C, 120 rpm. Cells were then harvested by centrifugation and resuspended in 500 μl PBS before analysis of cellular DHE fluorescence using a Beckman Astrios MoFlo cell sorter equipped with a 488-nm laser.

### Petite-cell formation.

Yeast cells were spread plated and grown for at least 7 days on YEPD agar, supplemented or not with 0.75 mM sorbic acid, and then colonies were replicated to fermentable (YEPD) or respiratory (YEPG) solid medium. After 3 days, the percentage of petite colonies was assessed according to respiratory deficiency (no growth) on YEPG versus YEPD. For the sorbic acid-supplemented YEPD (above), as low-pH agar degenerates with heating and will not subsequently set, agar medium at near-neutral pH was autoclaved before acidifying to pH 4.0 just before pouring. The YEPD was prepared without agar (not pH adjusted), and sorbic acid was added to defined concentrations. Samples (50 ml) were removed from each medium batch that contained sorbic acid and titrated to pH 4.0 with 5 M HCl to determine the volume of acid needed to adjust each batch to pH 4.0. Agar was added (16 g/liter) to the neutral non-pH-adjusted medium, which was then warmed to melt the agar before autoclaving. During subsequent cooling, the medium was held at 50°C, before acidification to pH 4.0 with the appropriate, predetermined volume of acid, and poured into petri dishes. The agar had no effect on pH or buffering.
