# Supplementary figures and images for: The Preservative Sorbic Acid Targets Respiration, Explaining the Resistance of Fermentative Spoilage Yeast Species
Source: mSphere. 2020 May 27;5(3):e00273-20. doi: 10.1128/mSphere.00273-20 (PMC7253596; doi:10.1128/mSphere.00273-20)

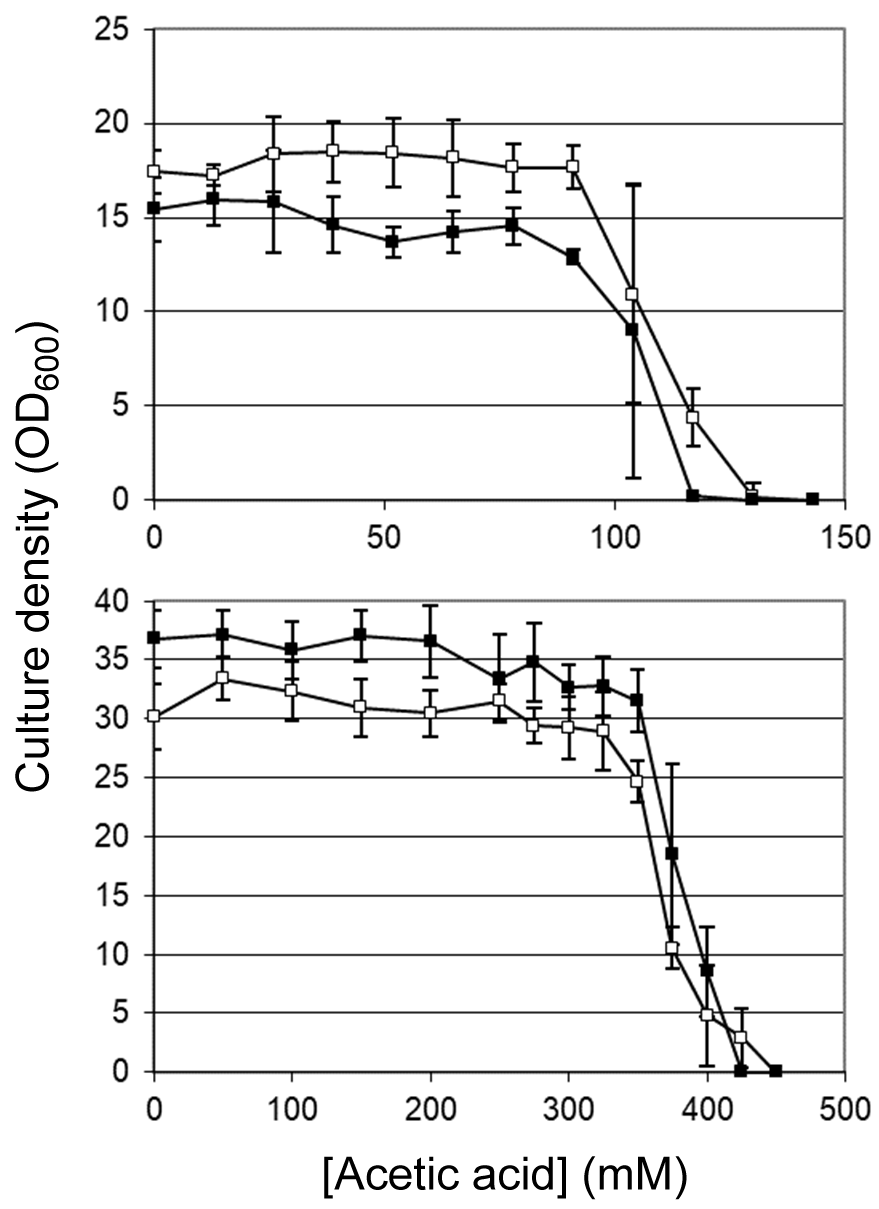

Supplement: FIG S1 [file mSphere.00273-20-sf001.tif]
