# Supplementary material for: The Preservative Sorbic Acid Targets Respiration, Explaining the Resistance of Fermentative Spoilage Yeast Species
Source: mSphere. 2020 May 27;5(3):e00273-20. doi: 10.1128/mSphere.00273-20 (PMC7253596; doi:10.1128/mSphere.00273-20)
Supplement: TABLE S1 [file mSphere.00273-20-st001.doc]

| **Strain** | **Previous Species** | **Current Species** | **Origin** |
| --- | --- | --- | --- |
| NY40 | *Aureobasidium pullulans* | *Aureobasidium pullulans* | Factory airplate UK |
| 69 | *Candida parapsilosis* | *Candida parapsilosis* | Spoilage, fruit juice |
| 519 | *Candida pseudointermedia* | *Candida pseudointermedia* | Factory floor, Brazil |
| NCYC 3297 | *Candida pseudolambica* | *Candida pseudolambica* | Factory drain, Brazil |
| 546 | *Cryptococcus laurentii* | *Cryptococcus laurentii* | Factory drain, Brazil |
| 628 | *Cryptococcus magnus* | *Cryptococcus magnus* | Scrapper Factory, Russia |
| 522 | *Issatchenkia orientalis* | *Pichia kudriavzevii* | Factory drain, Brazil |
| NCYC 3371 | *Pichia anomala* | *Wicherhamomyces anomalus* | Factory, Israel |
| 92 | *Rhodotorula glutinis* | *Rhodotorula glutinis* | Factory, Israel |
| 95 | *Rhodotorula mucilaginosa* | *Rhodotorula mucilaginosa* | Factory wall, UK |
| NCYC 3368 | *Saccharomyces cerevisiae* | *Saccharomyces cerevisiae* | Wine yeast |
| BY4741 | *Saccharomyces cerevisiae* | *Saccharomyces cerevisiae* | Laboratory strain |
| BY4741 Δ*pad1* | *Saccharomyces cerevisiae* | *Saccharomyces cerevisiae* | Laboratory strain |
| BY4743 | *Saccharomyces cerevisiae* | *Saccharomyces cerevisiae* | Laboratory strain |
| BY4743 Δ*bol3* | *Saccharomyces cerevisiae* | *Saccharomyces cerevisiae* | Laboratory strain |
| BY4743 Δ*grx5* | *Saccharomyces cerevisiae* | *Saccharomyces cerevisiae* | Laboratory strain |
| BY4743 Δ*iba57* | *Saccharomyces cerevisiae* | *Saccharomyces cerevisiae* | Laboratory strain |
| BY4743 Δ*isu1* | *Saccharomyces cerevisiae* | *Saccharomyces cerevisiae* | Laboratory strain |
| BY4743 Δ*nfu1* | *Saccharomyces cerevisiae* | *Saccharomyces cerevisiae* | Laboratory strain |
| BY4743 Δ*ssq1* | *Saccharomyces cerevisiae* | *Saccharomyces cerevisiae* | Laboratory strain |
| 55 | *Saccharomyces exiguus* | *Kazachstania exigua* | Spoilage, mayonnaise salad |
| 529 | *Torulaspora delbruckii* | *Torulaspora delbruckii* | Factory filler, Brazil |
| 405 | *Trichosporon ovoides* | *Trichosporon ovoides* | Factory pallet, Turkey |
| NCYC 1766 | *Zygosaccharomyces bailii* | *Zygosaccharomyces bailii* | Spoilage, fruit juice |
| NCYC 1555 | *Zygosaccharomyces bisporus* | *Zygosaccharomyces bisporus* | Spoilage, salad cream |
| NCYC 2789 | *Zygosaccharomyces lentus* | *Zygosaccharomyces lentus* | Spoilage, orange juice |
