# Supplementary material for: The Preservative Sorbic Acid Targets Respiration, Explaining the Resistance of Fermentative Spoilage Yeast Species
Source: mSphere. 2020 May 27;5(3):e00273-20. doi: 10.1128/mSphere.00273-20 (PMC7253596; doi:10.1128/mSphere.00273-20)
Supplement: TABLE S3 [file mSphere.00273-20-st003.doc]

|  | | | **Capacity to** | **Glucose fermentation (mls/ml)** | |
| --- | --- | --- | --- | --- | --- |
| **Group^a^** | **Strain** | **Yeast Species** | **Ferment^b^** | **20 g/l^c^** | **180 g/l** |
| 3 | 628 | *Cryptococcus magnus* | No | 0^d^ | 0 |
| 3 | 95 | *Rhodotorula mucilaginosa* | No | 0 | 0 |
| 3 | 92 | *Rhodotorula glutinis* | No | 0 | 0 |
| 3 | 546 | *Cryptococcus laurentii* | No | 0 | 0 |
| 2 | NCYC 3371 | *Wicherhamomyces anomalus* | Yes | 2.7 | 18.4 |
| 2 | 519 | *Candida pseudointermedia* | Yes | 2.8 | 11.6 |
| 2 | 69 | Candida parapsilosis | Yes | 2.8 | 9.6 |
| 2 | 529 | *Torulaspora delbruckii* | Yes | 3 | 34 |
| 2 | BY4741 | *Saccharomyces cerevisiae* | Yes | 3.2 | 34.5 |
| 2 | BY4741 Δ*pad1* | *Saccharomyces cerevisiae* | Yes | 3.3 | 36 |
| 2 | BY4741 petite | *Saccharomyces cerevisiae* | Yes | 3.1 | 35 |
| 1 | NCYC 3297 | *Candida pseudolambica* | Yes | 2.7 | 6.6 |
| 1 | 55 | *Kazachstania exigua* | Yes | 2.9 | 33.6 |
| 1 | 522 | *Pichia kudriavzevii* | Yes | 2.8 | 29 |
| 1 | NCYC 1555 | *Zygosaccharomyces bisporus* | Yes | 3.1 | 29.2 |
| 1 | NCYC 1766 | *Zygosaccharomyces bailii* | Yes | 3 | 36 |
| 1 | NCYC 2789 | *Zygosaccharomyces lentus* | Yes | 2.8 | 27.8 |

^a^Davenport grouping according to spoilage incidence (6). ^b^According to (1). ^c^Glucose concentration supplied. ^d^Fermentation was determined according to the gas pressure after 28 days at 24°C in replicate, static bottles in YEP supplemented with the indicated glucose concentration.
