# Supplementary material for: The Preservative Sorbic Acid Targets Respiration, Explaining the Resistance of Fermentative Spoilage Yeast Species
Source: mSphere. 2020 May 27;5(3):e00273-20. doi: 10.1128/mSphere.00273-20 (PMC7253596; doi:10.1128/mSphere.00273-20)
Supplement: TABLE S4 [file mSphere.00273-20-st004.doc]

|  | **Number of carbons** |  | **Weak acid MIC^a^ (mM)** | | **MIC ratio** |
| --- | --- | --- | --- | --- | --- |
|  |  | **cLogP** | **Glucose** | **Glycerol** | **Glyc/Gluc** |
| Acetic acid | 2 | -0.194 | 130 | 117 | 0.90 |
| Propionic acid | 3 | 0.335 | 60 | 50 | 0.83 |
| Butyric acid | 4 | 0.864 | 26 | 21 | 0.81 |
| Valeric acid | 5 | 1.393 | 9.25 | 7 | 0.76 |
| Hexanoic acid | 6 | 1.922 | 3.1 | 2.1 | 0.68 |
| Heptanoic acid | 7 | 2.451 | 1.65 | 0.9 | 0.55 |
| Octanoic acid | 8 | 2.98 | 0.99 | 0.55 | 0.53 |

^a^Weak acid MIC, mean from replicate determinations after 14 d shaking at 120 rev. min^-1^, 24°C in flasks containing YEP, pH 4.0 supplemented with weak acid and 30 g/l of either glucose or glycerol.
