# Supplementary material for: The Preservative Sorbic Acid Targets Respiration, Explaining the Resistance of Fermentative Spoilage Yeast Species
Source: mSphere. 2020 May 27;5(3):e00273-20. doi: 10.1128/mSphere.00273-20 (PMC7253596; doi:10.1128/mSphere.00273-20)
Supplement: TABLE S5 [file mSphere.00273-20-st005.doc]

| **ORF** | **Gene name** | **Function** |
| --- | --- | --- |
| YBR065C | ECM2 | Pre-mRNA splicing factor; facilitates the cooperative formation of U2/U6 helix II in association with stem II in the spliceosome, function may be regulated by Slu7 |
| YHR032W | ERC1 | Member of the multi-drug and toxin extrusion (MATE) family; the MATE family is part of the multidrug/oligosaccharidyl-lipid/polysaccharide (MOP) exporter superfamily; overproduction confers ethionine resistance and accumulation of S-adenosylmethionine |
| YGR263C | SAY1 | Sterol deacetylase; component of the sterol acetylation/deacetylation cycle along with Atf2p; active both in the endoplasmic reticulum (ER) and in lipid droplets; integral membrane protein with active site in the ER lumen; green fluorescent protein (GFP)-fusion protein localizes to the ER |
| YDL184C | RPL41A | Ribosomal 60S subunit protein L41A; comprises only 25 amino acids; rpl41a rpl41b double null mutant is viable; homologous to mammalian ribosomal protein L41, no bacterial homolog; RPL41A has a paralog, RPL41B, that arose from the whole genome duplication |
| YDR278C |  | Putative protein of unknown function; conserved among S. cerevisiae strains; YDR278C is not an essential gene |
| YBR076W | ECM8 | Non-essential protein of unknown function |
| YJR119C | JHD2 | JmjC domain family histone demethylase; promotes global demethylation of H3K4 and repression of noncoding intergenic transcription during sporulation; removes methyl groups added by Set1p; negatively regulated by H3K14 acetylation; protein levels regulated by Not4p polyubiquitin-mediated degradation; regulates sporulation timing by extending period of active transcription; regulates rDNA silencing; human homolog is JARID1C |
| YGL243W | TAD1 | tRNA-specific adenosine deaminase; deaminates adenosine-37 to inosine in tRNA-Ala |
| YLR448W | RPL6B | Ribosomal 60S subunit protein L6B; binds 5.8S rRNA; homologous to mammalian ribosomal protein L6, no bacterial homolog; RPL6B has a paralog, RPL6A, that arose from the whole genome duplication |
| YGR153W |  | Putative protein of unknown function |
| YKL066W |  | Dubious open reading frame; unlikely to encode a functional protein, based on available experimental and comparative sequence data; not conserved in closely related Saccharomyces species; partially overlaps the verified gene YNK1 |
| YHR184W | SSP1 | Protein involved in the control of meiotic nuclear division; involved in the coordination of meiosis with spore formation; subunit of the leading edge protein (LEP) complex (Ssp1-Ady3-Don1-Irc10) that forms a ring-like structure at the leading edge of the prospore membrane during meiosis II; required for assembly of the leading edge coat and both prospore membrane shaping and organization; transcription is induced midway through meiosis |
| YJR137C | MET5 | Sulfite reductase beta subunit; involved in amino acid biosynthesis, transcription repressed by methionine |
| YMR132C | JLP2 | Protein of unknown function; contains sequence that closely resembles a J domain (typified by the E. coli DnaJ protein) |
| YGL228W | SHE10 | Protein involved in outer spore wall assembly; likely involved directly in dityrosine layer assembly; putative GPI-anchored protein; overexpression causes growth arrest;; SWAT-GFP, seamless-GFP and mCherry fusion proteins localize to the endoplasmic reticulum; SHE10 has a paralog, OSW7/YFR039C, that arose from the whole genome duplication; paralogs are redundant for spore wall dityrosine assembly |
| YGR196C | FYV8 | Protein of unknown function; required for survival upon exposure to K1 killer toxin |
| YJR121W | ATP2 | Beta subunit of the F1 sector of mitochondrial F1F0 ATP synthase; which is a large, evolutionarily conserved enzyme complex required for ATP synthesis; F1 translationally regulates ATP6 and ATP8 expression to achieve a balanced output of ATP synthase genes encoded in nucleus and mitochondria; phosphorylated |
| YDR015C |  | Dubious open reading frame; unlikely to encode a functional protein, based on available experimental and comparative sequence data; overlaps the verified gene HED1/YDR014W-A |
| YGR283C |  | Putative methyltransferase; may interact with ribosomes, based on co-purification experiments; predicted to be involved in ribosome biogenesis; null mutant is resistant to fluconazole; GFP-fusion protein localizes to the nucleolus; YGR283C has a paralog, YMR310C, that arose from the whole genome duplication |
| YLR209C | PNP1 | Purine nucleoside phosphorylase; specifically metabolizes inosine and guanosine nucleosides; involved in the nicotinamide riboside salvage pathway |
| YBR099C |  | Dubious open reading frame; unlikely to encode a functional protein, based on available experimental and comparative sequence data; completely overlaps the verified gene MMS4 |
| YCL026C |  | Deleted ORF; does not encode a protein; included in the original annotation of Chromosome III but later deleted due to sequence corrections |
| YGL253W | HXK2 | Hexokinase isoenzyme 2; phosphorylates glucose in cytosol; predominant hexokinase during growth on glucose; represses expression of HXK1, GLK1, induces expression of its own gene; antiapoptotic; phosphorylation/dephosphorylation at Ser14 by kinase Snf1p, phosphatase Glc7p-Reg1p regulates nucleocytoplasmic shuttling of Hxk2p; functions downstream of Sit4p in control of cell cycle, mitochondrial function, oxidative stress resistance, chronological lifespan; has paralog HXK1 |
| YLR388W | RPS29A | Protein component of the small (40S) ribosomal subunit; homologous to mammalian ribosomal protein S29 and bacterial S14; RPS29A has a paralog, RPS29B, that arose from the whole genome duplication |
| YMR107W | SPG4 | Protein required for high temperature survival during stationary phase; not required for growth on nonfermentable carbon sources |
| YDL149W | ATG9 | Transmembrane protein involved in forming Cvt and autophagic vesicles; cycles between the phagophore assembly site (PAS) and other cytosolic punctate structures, not found in autophagosomes; may be involved in membrane delivery to the PAS |
| YDR094W |  | Dubious open reading frame; unlikely to encode a functional protein, based on available experimental and comparative sequence data; partially overlaps verified ORF DNF2/YDR093W |
| YJL047C | RTT101 | Cullin subunit of a Roc1p-dependent E3 ubiquitin ligase complex; role in anaphase progression; required for recovery after DSB repair; implicated in Mms22-dependent DNA repair; involved with Mms1p in nonfunctional rRNA decay; modified by the ubiquitin-like protein, Rub1p |
| YMR214W | SCJ1 | One of several homologs of bacterial chaperone DnaJ; located in the ER lumen where it cooperates with Kar2p to mediate maturation of proteins |
| YDL080C | THI3 | Regulatory protein that binds Pdc2p and Thi2p transcription factors; activates thiamine biosynthesis transcription factors Pdc2p and Thi2p by binding to them, but releases and de-activates them upon binding to thiamine pyrophosphate (TPP), the end product of the pathway; has similarity to decarboxylases but enzymatic activity is not detected |
| YDL186W |  | Putative protein of unknown function; YDL186W is not an essential gene |
| YHR104W | GRE3 | Aldose reductase; involved in methylglyoxal, d-xylose, arabinose, and galactose metabolism; stress induced (osmotic, ionic, oxidative, heat shock, starvation and heavy metals); regulated by the HOG pathway; protein abundance increases in response to DNA replication stress |
| YHR117W | TOM71 | Mitochondrial outer membrane protein; probable minor component of the TOM (translocase of outer membrane) complex responsible for recognition and import of mitochondrially directed proteins; TOM71 has a paralog, TOM70, that arose from the whole genome duplication |
| YLR318W | EST2 | Reverse transcriptase subunit of the telomerase holoenzyme; essential for telomerase core catalytic activity, involved in other aspects of telomerase assembly and function; mutations in human homolog are associated with aplastic anemia |
| YLR367W | RPS22B | Protein component of the small (40S) ribosomal subunit; homologous to mammalian ribosomal protein S15A and bacterial S8; RPS22B has a paralog, RPS22A, that arose from the whole genome duplication |

^a^a preliminary screen of the *S. cerevisiae* yeast deletant collection (homozygous diploid) on YEP-glycerol, pH 4.0 indicated 126 mutants with improved growth on sorbic acid versus wild type. Further testing of these hits refined the list of resistant mutants to those listed in the table, which details the relevant (deleted) gene functions. Listed strains had a growth ratio significantly greater (p<0.05, t-test) than the wild type, where growth ratio = colony area sorbic acid / colony area control.
